# Gene therapy for disorders of sex development: current applications and future challenges

**DOI:** 10.3389/fgene.2025.1661127

**Published:** 2025-10-28

**Authors:** Wenyuan Peng, Qian Zhao, Jiali Chen, Huifang Peng, Hongwei Jiang

**Affiliations:** Henan Key Laboratory of Rare Diseases, Endocrinology and Metabolism Center, The First Affiliated Hospital, and College of Clinical Medicine of Henan University of Science and Technology, Luoyang, China

**Keywords:** DSD, 46,XY DSD, 46,XX DSD, gene therapy, sex determination

## Abstract

Disorders of sex development (DSD) represent a spectrum of congenital conditions where discrepancies exist between chromosomal, gonadal, or anatomical sex. Recent advances in genomic diagnostics and gene-editing technologies have enabled significant progress in the identification of pathogenic variants and the exploration of targeted therapeutic strategies. This review systematically examines the roles of key sex-determining genes—including SRY, SOX9, NR5A1, WT1, FOXL2, and AR—in various DSD subtypes. It further elaborates on gene therapy strategies targeting these loci through the use of CRISPR/Cas9, TALENs, ZFNs, and viral vector-mediated delivery systems. Notably, CRISPR/Cas9 has been utilized to correct or epigenetically activate gene expression *in vitro*, such as SRY promoter demethylation in embryonic stem cells, and targeted disruption of SOX9 enhancers to model 46, XX testicular DSD in mice. Additionally, lentiviral vectors have enabled stable overexpression of transcriptional regulators (e.g., SOX9, NR5A1) in hiPSCs, inducing differentiation into Sertoli- and Leydig-like cells, with partial restoration of testicular function *in vitro*. Complementarily, AAV-based vectors—including AAV8 and synthetic AAVDJ—have demonstrated effective delivery of genes like Lhcgr into testicular interstitial tissues, restoring testosterone synthesis and fertility in mouse models. Despite this progress, current gene therapy approaches still face considerable technical challenges, such as off-target effects, immunogenicity of viral vectors or editing enzymes, and long-term transgene expression instability. Germline editing, while theoretically advantageous for early-onset DSD phenotypes, introduces profound ethical dilemmas due to its heritable nature. These include concerns regarding informed consent in minors, gender identity autonomy, and societal consequences of altering reproductive cells. Current international bioethics frameworks urge caution and recommend limiting clinical applications to somatic cells under stringent regulatory oversight. In conclusion, gene therapy offers a transformative potential for the diagnosis and treatment of DSD. Future directions should prioritize enhanced safety, precision delivery systems, and an ethically guided clinical translation pathway to ensure long-term efficacy and societal acceptability.

## 1 Introduction

Disorder of sex development (DSD) encompass a spectrum of congenital conditions characterized by atypical chromosomal, gonadal, or anatomical sexual differentiation (Raza et al., 2019). These disorders arise from heterogeneous etiologies involving both genetic mutations (e.g., variants in sex-determining region of Y-chromosome, SRY; nuclear receptor subfamily 5 group A member 1, NR5A1; SRY-Box Transcription Factor 9, SOX9) and environmental influences (e.g., prenatal hormone exposure) (Raza et al., 2019). Advances in genomic technologies—including next-generation sequencing (NGS) and CRISPR-based screens—have exponentially expanded the catalog of DSD-associated genes, accurate diagnosisand enabling personalized therapeutic strategies (Raza et al., 2019; Stancampiano et al., 2021).

Gene therapy has demonstrated transformative success in monogenic disorders such as spinal muscular atrophy (SMA) and Leber congenital amaurosis, while the application in DSD treatment has only seen a few experimental explorations (Islam et al., 2019). This review delineates the molecular underpinnings of DSD pathogenesis, evaluates the current landscape of gene-editing, gene replacement, and epigenetic modulation therapies, and critically addresses persistent challenges—including off-target effects, immune responses, and ethical considerations. Furthermore, we explore emerging paradigms such as *in vivo* reprogramming and organoid-based models that may redefine future DSD therapeutics.

## 2 The key genes and molecular mechanisms of DSD

### 2.1 SRY in sex development

The SRY gene is located on the short arm of the Y chromosome (Yp11.3), spanning approximately 828 base pairs (bp) in the DNA sequence and encoding a protein composed of 204 amino acids (https://www.uniprot.org/uniprotkb/Q05066/entry).The SRY protein contains a highly conserved High Mobility Group (HMG) domain, which facilitates binding to DNA and induces DNA bending, thereby regulating the expression of downstream target genes (Bashamboo and McElreavey, 2016). As the master regulator of male sex determination, SRY plays a pivotal role in initiating testicular development during embryogenesis. Its expression is activated around weeks 6–8 of gestation, during which it upregulates SOX9 and other testis-determining factors, driving testicular differentiation (Bashamboo and McElreavey, 2016; Assumpção et al., 2005). Loss of function or aberrant expression of the SRY gene may lead to male-to-female sex reversal, even in individuals carrying a Y chromosome, thus resulting in DSD (Assumpção et al., 2005).

In a study, researchers pointed out that there are multiple Sp1 binding sites in the SRY promoter region. Sp1 is a widely present zinc finger transcription factor that can bind to GC-rich regions on DNA, thereby regulating the transcription of various genes. In the regulation of SRY expression, Sp1 enhances transcriptional activity by directly binding to its promoter region. One of these sites is located between c.-130 and c.-124. The absence or mutation of this site can affect Sp1 binding, leading to insufficient SRY gene expression, which in turn affects the process of sex determination and can result in the occurrence of DSD (Assumpção et al., 2005). During human embryogenesis (weeks 6–7 postconception), SRY protein directly binds to the promoter of SOX9 gene, initiating its expression to drive testis formation. Pathogenic SRY mutations or deletions in 46, XY individuals result in Swyer syndrome (46, XY complete gonadal dysgenesis, CGD).

Recent cohort-based genetic studies have reaffirmed the pivotal role of SRY gene in 46, XX testicular DSD and 46, XY complete gonadal dysgenesis (CGD). A comprehensive multicenter cohort study by Berglund et al. published in Biology of Sex Differences analyzed a group of phenotypically male individuals with 46, XX karyotype and performed genomic, transcriptomic, and Y-chromosome segment length analyses. Among the 46, XX DSD patients, the presence of the SRY gene was confirmed in all but one individual, indicating that SRY translocation to an autosome or X chromosome remains the most common genetic mechanism for testicular development in these individuals. Interestingly, this study also revealed that SRY-positive patients exhibited considerable heterogeneity in the size and gene content of Y-chromosome-derived material, highlighting variability in gene dosage effects and secondary modifying loci (Berglund et al., 2024). In parallel, Tadokoro-Cuccaro and Hughes published an extensive phenotype-genotype analysis of individuals with 46, XY CGD and partial gonadal dysgenesis (PGD). In this study, SRY mutations were identified as one of the most frequent causes of CGD, along with WT1 variants. Among the cohort, 42% had a defined genetic cause, underscoring the importance of including SRY sequencing and dosage assessment in routine diagnostic pipelines for DSD evaluation (Tadokoro-Cuccaro et al., 2025). Furthermore, a large-scale epidemiological study in Iran also screened SRY gene status in 46, XX DSD individuals and found that approximately 85% of testicular DSD patients carried a translocated SRY, while the remainder were SRY-negative, prompting investigations into SOX9 upregulation and other autosomal modifiers (Rastari et al., 2025).

### 2.2 SOX9 in sex development

The SOX9 gene is located at chromosome 17q24.3. The gene spans approximately 1.1 million base pairs, although the exact length varies depending on transcript variants. The main transcript is approximately 2.8 kb and encodes a protein of 509 amino acids (https://www.uniprot.org/uniprotkb/P48436/entry). This protein contains a HMG-box DNA-binding domain, which is highly homologous to that of SRY and consists of 79 amino acid residues. The HMG-box domain binds to the minor groove of DNA, inducing DNA bending of approximately 70–90°. Compared to the SRY HMG-box, which preferentially binds to A/T-rich minor grooves and exhibits transient interactions, the HMG-box of SOX9 tends to form more stable DNA–protein complexes, enabling sustained gene regulation. This allows SOX9 to maintain long-term expression and regulate multiple downstream targets (such as Anti-Müllerian Hormone [AMH]) through positive feedback loops, thereby modulating transcriptional activity (Vining et al., 2021). The subcellular localization of SOX9 is the nucleus, where it functions as a transcription factor to regulate the expression of multiple genes involved in sex differentiation, chondrogenesis, and organogenesis (https://www.uniprot.org/uniprotkb/P48436/entry) (Yavas Abalı and Guran, 2024).

The SOX9 gene plays a critical role in testis development and male sex determination in mammals, whose expression is directly activated by SRY, initiating a cascade of downstream gene activations essential for testicular differentiation. Among these targets, AMH is particularly important. Secreted by Sertoli cells, AMH induces regression of the Müllerian ducts, thereby preventing the formation of female reproductive structures. In addition to AMH, SOX9 upregulates Fibroblast Growth Factor 9 (FGF9), Desert Hedgehog (DHH), and Prostaglandin D2 Synthase (PTGDS). These factors contribute to testis cord formation, recruitment of interstitial cells, and reinforcement of SOX9 expression through positive feedback loops. These molecular circuits ensure the sustained expression of SOX9 and repression of the ovarian pathway, thereby stabilizing the development of the male gonads during early embryogenesis.[11] In mice, embryonic day 10.5 (E10.5) marks a critical time point for the onset of SOX9 expression, typically occurring within 24 h after SRY activation, highlighting the rapid and essential process of SOX9 initiation. In humans, SOX9 is upregulated during embryonic weeks 6–8, which coincides with the onset of gonadal differentiation. SOX9 maintains high expression levels through the feedback network with FGF9 and PGD2, ensuring stable testis formation (Vining et al., 2021).

In a cohort of 46, XX individuals, Researchers have highlighted that, duplications involving the SOX9 gene or its upstream regulatory regions (e.g., enhancer regions located approximately 600 kb upstream) can still drive the formation of testicular or testis-like tissue, thereby inducing male phenotypic features or DSD in 46, XX individuals. This finding underscores that dysregulated SOX9 expression can bypass the SRY pathway and independently initiate testicular developmental programs (Yavas Abalı and Guran, 2024).

### 2.3 NR5A1 (SF1) in sex development

The NR5A1 (Steroidogenic factor 1, SF1) gene is located in the 9q33.3, spanning approximately 30 kb and containing 7 exons. Its transcript is 2,842 bp in length, encoding a 461-amino acid nuclear transcription factor (molecular weight ∼52.6 kDa) (https://www.uniprot.org/uniprotkb/Q13285/entry). This protein contains critical DNA-binding (DBD, with zinc finger motifs) and ligand-binding (LBD) domains, and regulates genes involved in gonadal and adrenal development by binding to target gene promoters (e.g., the AGGTCA sequence). NR5A1 is localized to the nucleus and plays a role in the early stages of gonadal differentiation (expression begins at human embryonic weeks 4–5). During sex determination, NR5A1 may enhance the transcriptional activity of SRY and SOX9, promoting Sertoli cell differentiation (https://www.uniprot.org/uniprotkb/Q13285/entry) (Eggers et al., 2016).

Mutations in NR5A1 (e.g., 3-bp deletions or missense mutations like p. Arg92Trp) frequently occur in the DBD or LBD, leading to 46, XY DSD (gonadal dysgenesis, hypospadias, adrenal insufficiency in some cases) or 46, XX testicular or ovotesticular DSD. NR5A1 cooperates with SRY and SOX9, binding regulatory regions of target genes, like the Sox9 promoter/enhancer in mice, to activate the gene expression program for gonadal differentiation (Sekido and Lovell-Badge, 2008).

Recent cohort studies have solidified the NR5A1 gene as a major contributor to 46, XY DSD, particularly in individuals presenting with PGD or undervirilization. A pivotal multicenter study by Naamneh-Elzenaty, known as the SF1next cohort, characterized 35 novel NR5A1/SF-1 variants across 39 patients with atypical sexual development, confirming that mutations spanned both the DNA-binding domain and the ligand-binding domain of the SF-1 protein. Phenotypes ranged from ambiguous genitalia and cryptorchidism to complete gonadal dysgenesis, often without adrenal insufficiency—highlighting the variable penetrance and expressivity of NR5A1 mutations (Naamneh Elzenaty et al., 2025). Similarly, a global genetic cohort involving over 400 individuals with 46, XY DSD from Asia, Africa, and Europe revealed that NR5A1 was among the top three most frequently mutated genes, alongside AR and SRD5A2. Importantly, geographic and ethnic differences were noted in mutation frequency and clinical severity (Jiali et al., 2024).

In China, Chen applied targeted NGS to a large 46, XY DSD cohort and reported that NR5A1 variants accounted for a significant proportion of patients, particularly those with milder phenotypes and intact adrenal function. Notably, variants in codon R92 were recurrent and implicated in both XY and XX testicular DSD, suggesting a unique dosage-sensitive role of SF-1 in human gonadal differentiation (Chen et al., 2024).

### 2.4 Wilms tumor protein (WT1) gene in sex development

WT1 is located on 11p13 and comprises approximately 10 exons, with a total DNA length of around 50 kb. The WT1 protein consists of 449 amino acid residues and has a molecular weight of approximately 49,875 Da (∼49.8 kDa) (https://www.uniprot.org/uniprotkb/P19544/entry). Structurally, WT1 contains two main functional domains: a proline- and glutamine-rich N-terminal domain involved in transcriptional regulation, and a C-terminal domain comprising four C2H2-type zinc finger motifs, which confer sequence-specific DNA-binding capacity. WT1 is primarily localized in the cell nucleus which contains a well-defined nuclear localization signal (NLS) and binds directly to specific DNA sequences via its zinc finger domains. Depending on alternative splicing variants and interacting cofactors, WT1 can act as either a transcriptional activator or repressor, modulating the expression of various target genes, including IGF2, BCL2, and EGR1 (https://www.uniprot.org/uniprotkb/P19544/entry). Functionally, WT1 is a pleiotropic protein with essential roles in the development of the kidneys, gonads, and heart. It participates in embryonic development, cell proliferation, and apoptosis, and serves as a classical tumor suppressor. Mutations in WT1 are implicated in the pathogenesis of Wilms tumor and several forms of DSD (https://www.uniprot.org/uniprotkb/P19544/entry).

Recent cohort-based investigations have highlighted the central role of WT1 gene mutations in the pathogenesis of 46, XY DSD and 46, XX testicular DSD (Sirokha et al., 2021), particularly in cases of partial gonadal dysgenesis (PGD). A 2025 prospective multicenter cohort study led by Tadokoro-Cuccaro and Hughes analyzed 12 individuals with 46, XY PGD, revealing that 83% (10/12) carried pathogenic variants in WT1, most frequently located in exons encoding the fourth zinc finger domain, which is essential for DNA binding and transcriptional regulation of sex-determining genes (Tadokoro-Cuccaro et al., 2025). These variants correlated with variable phenotypes, including ambiguous genitalia, cryptorchidism, and delayed or incomplete puberty, underscoring the phenotypic heterogeneity of WT1-related DSD. Another large-scale genotype-phenotype correlation study conducted in North Africa utilized WES in children with 46, XY DSD. It confirmed that WT1 mutations were present in a significant subset of patients, some of whom also developed bilateral Wilms tumors, highlighting the dual role of WT1 in gonadal and renal development (Wong et al., 2025). Furthermore, a 2024 Iranian cohort study reported that variants within the fourth zinc finger region of WT1 were consistently associated with severe gonadal dysgenesis and an increased risk of gonadoblastoma. This study was the first to systematically describe DSD at a national level and showed that WT1 variants were among the top three monogenic causes (Rastari et al., 2025).

### 2.5 FOXL2 gene in sex development

The FOXL2 gene is located on 3q23, approximately 2.9 kb (2,900 bp), including a main exon coding region. The primary transcript length is about 2.1 kb, encoding 376 amino acids, with a protein molecular weight of about 42.3 kDa, containing the classic Forkhead DNA-binding domain (i.e., “winged helix structure”), located in the middle region of the protein (approximately amino acids 52-152) (https://www.uniprot.org/uniprotkb/P58012/entry). Its subcellular localization is the cell nucleus, with a typical NLS. FOXL2 is a key transcription factor in the development and maintenance of the ovaries; it regulates multiple target genes (such as CYP19A1, StAR, GDF9), and its core function is determined by the Forkhead domain, which can specifically bind to the conserved sequences in the promoter regions of target genes. Its functions include positively regulating the expression of ovarian-related genes (CYP19A1, BMP15); suppressing the expression of testis gene pathways (SOX9); maintaining the differentiation and function of granulosa cells; recruiting coactivator or corepressor complexes, such as SMADs, HDACs, etc (https://www.uniprot.org/uniprotkb/P58012/entry). FOXL2 can directly or indirectly inhibit the expression of SOX9, a key determinant of testis development, thereby preventing the formation of testicular tissue during female gonad development. Additionally, FOXL2 regulates critical reproductive hormone-related genes, such as CYP19A1 (aromatase), promoting estrogen synthesis (Veitia, 2010).

In a study led by Chen, researchers performed targeted gene panel sequencing on 402 Chinese patients diagnosed with 46, XY DSD. The customized panel included a wide range of common DSD-related genes, among which FOXL2 was incorporated for systematic screening of potential pathogenic variants (Chen et al., 2024). The results demonstrated that mutations in the FOXL2 gene were rare among the 46, XY DSD cohort and were not listed among the top five most frequent pathogenic genes. However, FOXL2-related variants still accounted for a proportion of the total variants of uncertain significance (VUS), particularly concentrated within the coding region and the 5′regulatory sequences (Chen et al., 2024). Given the crucial transcriptional regulatory role of FOXL2 in embryonic gonadal differentiation, the authors hypothesized that even in 46, XY individuals, aberrations in FOXL2 might indirectly contribute to disordered sex development by impacting the differentiation of gonadal support cells. Nonetheless, the specific mechanisms underlying this association require further functional validation. The study emphasized that within the VUS category, it remains challenging to determine the pathogenicity of FOXL2 variants based solely on sequencing data. Additional verification through *in vitro* cellular models or *in vivo* animal experiments is necessary (Chen et al., 2024).

### 2.6 Androgen receptor (AR) gene in sex development

AR is located on chromosome Xq11–12. The full gene spans approximately 90 kb and contains eight exons. Its primary transcript is about 8.9 kb in length, encoding a protein composed of 919 amino acids with a molecular weight of approximately 98 kDa. The AR protein comprises three major functional domains: the N-terminal transactivation domain (NTD), which regulates transcriptional activity; the DBD, which contains two C4-type zinc finger motifs; and the LBD, which binds to testosterone or dihydrotestosterone (https://www.uniprot.org/uniprotkb/P10275/entry). Subcellularly, AR is initially localized in the cytoplasm, and upon ligand binding, it translocates to the nucleus to execute its transcriptional regulatory functions. AR belongs to the nuclear receptor superfamily of transcription factors. Once activated by androgen ligands, it initiates the transcription of target genes involved in male sex differentiation, spermatogenesis, and prostate development. Mutations in the AR gene are a major cause of both complete androgen insensitivity syndrome (CAIS) and partial androgen insensitivity syndrome (PAIS), which represent key phenotypic manifestations of 46, XY DSD (https://www.uniprot.org/uniprotkb/P10275/entry; https://www.ncbi.nlm.nih.gov/gene/367).

A large-scale cohort study led by Chen conducted targeted gene panel sequencing on 402 Chinese patients with 46, XY DSD. The overall diagnostic yield was 11.2% (45 cases). Although the AR gene was not among the most commonly identified pathogenic genes, it was included as a classical DSD-related gene in the validated screening panel. Additionally, 15.4% of patients (62 cases) carried variants of uncertain significance (VUS), some of which were missense variants located within the DBD of AR. The rate of uncertain variants was even higher in sporadic cases (15.1%), indicating that interpreting AR variants remains challenging in the absence of family-based segregation analysis (Chen et al., 2024).

## 3 Gene therapy strategies in DSD: target-oriented approaches

Recent advances in gene therapy have introduced new strategies and methodologies for the treatment of DSD, particularly in the areas of gene editing, replacement strategies, and transcriptional regulation (Table 1). Here, we summarize the latest developments and assess their therapeutic potential in the management of DSD.

**TABLE 1:** Key genes and gene therapy strategies in DSD.

Gene	Associated disorders	Gene therapy strategy	Experimental model	Technology and vectors used	Mode of therapy
SRY	46, XX Testicular DSD; 46, XY Gonadal Dysgenesis	AAV-mediated SRY expression	46, XX DSD mouse model	AAV9 vector + CRISPR/Cas9	Gene replacement
SOX9	Campomelic Dysplasia; 46, XY Gonadal Dysgenesis	CRISPR-mediated repair + lentiviral SOX9 overexpression	Patient-derived iPSCs	LVSOX9 + CRISPR/Cas9	Gene activation
NR5A1	46, XY DSD; Adrenal Hypoplasia	AAV-mediated expression enhancement, CRISPR activation and repair	NR5A1-deficient mouse model	AAV8 vector + CRISPRa/Cas9-HDR	Activation/Correction
WT1	Denys-Drash Syndrome; Frasier Syndrome	CRISPR-mediated WT1 mutation correction	WT1 mutant mouse model	CRISPR/Cas12a + Lentivirus	Gene correction
FOXL2	46, XX Gonadal Dysgenesis; Blepharophimosis-Ptosis-Epicanthus Syndrome (BPES)	Expression regulation; enhancer knockout	FOXL2-mutant DSD mouse model	CRISPR/Cas9 + AAVDJ	Gene silencing
AR	Androgen Insensitivity Syndrome (AIS)	CRISPR-mediated exon correction + ASO-mediated silencing	AIS patient-derived cell lines, iPSCs	CRISPR (RNA or DNA) + Antisense Oligonucleotides	Gene correction

### 3.1 Targeted modulation of sex-determining genes using CRISPR/Cas9 in DSD

In recent years, the CRISPR/Cas9 system, a highly specific and efficient genome-editing tool, has shown considerable promise in the investigation of molecular mechanisms and therapeutic strategies for DSD. Numerous studies have applied this technology to key sex-determining genes—such as SRY, SOX9, and NR5A1—targeting their promoters, enhancers, and coding regions, with the aim of elucidating regulatory pathways and constructing potential intervention models.

Regarding the SRY gene, Okashita designed guide RNAs (gRNAs) targeting the upstream promoter region (approximately −300 bp to the transcription start site) and used CRISPR/Cas9 to induce double-stranded DNA breaks in mouse embryonic stem cells to simulate local demethylation. Furthermore, the study employed siRNA-mediated knockdown of TET2, a DNA demethylase, and found that SRY expression was markedly reduced—even when promoter methylation levels were decreased—highlighting that SRY transcriptional activation via CRISPR editing is dependent on endogenous TET2 activity. This was the first study to demonstrate that CRISPR-mediated promoter editing requires epigenetic co-factors to activate SRY expression, providing direct evidence for the role of promoter demethylation in sex determination regulation (Okashita et al., 2019).

SOX9, a critical downstream effector in the sex determination cascade, is regulated by upstream enhancer elements such as Enh13 and Enh22. Croft et al. used CRISPR to delete the Enh13 region in mouse embryos and observed a significant reduction in SOX9 expression along with gonadal development failure, thereby mimicking the phenotype observed in 46, XX sex reversal patients (Croft et al., 2018). Building on this, Ridnik et al. carried out precise site-specific editing using two gRNAs targeting transcription factor binding sites for SRY and NR5A1 within Enh13. Their results revealed that disruption of either site alone had a limited effect, but simultaneous deletion of both led to complete abrogation of SOX9 transcription, ultimately resulting in sex determination failure and gonadal dysgenesis. This study provided critical insights into the redundant and cooperative functions of enhancer-bound transcription factors and supported the rationale for multiplexed CRISPR-based strategies in future DSD gene therapies (Ridnik et al., 2024).

For NR5A1, Gonen employed both CRISPRa (CRISPR activation) and CRISPR/Cas9-mediated repair strategies in a human induced pluripotent stem cell (hiPSC) model. In cells with reduced NR5A1 expression but without coding mutations, a dead Cas9-VP64 fusion was used to target the NR5A1 promoter, leading to significant upregulation of transcription and directed differentiation into gonadal support cell lineages expressing AMH and INSL3 (Gonen et al., 2023). In parallel, for models carrying pathogenic missense mutations (e.g., R92Q), the team utilized homology-directed repair (HDR) in combination with single-stranded oligonucleotide (ssODN) repair templates to correct the mutations. The corrected hiPSC-derived cells demonstrated functional characteristics of testicular support cells, including blood-testis barrier (BTB) properties and androgen biosynthesis capacity. Similarly, Danti et al. confirmed that NR5A1 activation in a human embryonic stem cell (hESC) system could direct bipotential gonadal-like cells toward a steroidogenic fate, supporting the therapeutic potential of targeted NR5A1 regulation in DSD (Danti et al., 2023).

### 3.2 Precision editing of point mutations and regulatory elements using TALENs and ZFNs in DSD

With the continuous advancement of genome editing technologies, TALENs and ZFNs, as early-generation precision genome editing tools, have demonstrated certain potential for application in the treatment of inherited diseases such as DSD. Both TALENs and ZFNs rely on artificially engineered DNA-binding modules to specifically recognize target sequences and utilize nucleases (typically FokI) to induce DNA double-strand breaks (DSBs). Subsequently, cellular repair mechanisms such as non-homologous end joining (NHEJ) or homology-directed repair (HDR) can lead to gene knockout, mutation correction, or targeted sequence insertion (Carroll, 2011).

Compared with the recently emerged CRISPR/Cas9 system, TALENs and ZFNs have the following characteristics: They offer higher sequence specificity and lower off-target rates, making them particularly suitable for precise corrections near single-nucleotide mutations (Urnov et al., 2010); They do not rely on the presence of protospacer adjacent motif (PAM) sequences, theoretically allowing targeting of any DNA region. However, they also present several disadvantages: Construction is complex and time-consuming, requiring custom protein engineering; Transfection efficiency can be limited by the relatively large molecular size; Editing efficiency at multiple sites is significantly lower compared to CRISPR systems.

Currently, the application of TALENs and ZFNs in DSD-related research mainly focuses on the following areas: First, the establishment of single-nucleotide mutation correction models.

Given that point mutations are common among DSD patients (such as inactivating mutations in the SRY gene, missense variants in NR5A1, and single-nucleotide mutations in FOXL2), TALENs and ZFNs are particularly suitable for precise correction of such point mutations. For example, Gonen utilized the TALEN system to repair microdeletions in regulatory elements upstream of the Sox9 gene (such as the Enh13 enhancer deletion model) in mice, successfully restoring gonadal support cell function, thereby demonstrating the potential application value of this technology in point mutation-based DSD models (Gonen et al., 2023). Second, functional validation of the regulatory networks of SRY and SOX9. Some foundational studies have employed TALEN-mediated knockout or modification of the SRY promoter and SOX9 enhancers (e.g., Enh13, Enh22) to investigate their roles during sex determination. Precise editing of these regulatory elements enables the modeling of gene dosage anomalies or promoter dysfunction observed in DSD patients, providing reliable experimental systems for the study of disease mechanisms.

### 3.3 AAV-mediated gene delivery for leydig cell and germline correction in DSD

AAV is a single-stranded DNA viral vector that has gained widespread use in gene therapy owing to its excellent biosafety profile, low immunogenicity, and broad tissue tropism (Naso et al., 2017). Unlike other integrating viral vectors, AAV predominantly remains episomal in the nucleus, minimizing the risk of insertional mutagenesis (Samulski and Muzyczka, 2014). Furthermore, advancements in self-complementary AAV (scAAV) have significantly enhanced transduction efficiency by bypassing the rate-limiting step of second-strand DNA synthesis (McCarty, 2008). These features make AAV highly suitable for *in vivo* gene delivery, especially in tissues with low proliferative indices such as the testes (Watanabe et al., 2018).

In comparative studies of AAV serotypes, AAV1, AAV8, and AAV9 have consistently demonstrated high transduction efficiency in testicular tissues; however, they differ significantly in terms of cell-type specificity and tissue penetrance. In a systematic study conducted by Watanabe the transduction profiles of multiple AAV serotypes were evaluated via direct intratesticular injection in mice, assessing their efficiency in targeting both germ cells and supporting somatic cells. This work provided key experimental evidence for the use of AAV-mediated gene delivery in the male germline system (Watanabe et al., 2018). The results showed that both AAV1 and AAV9 were capable of effectively penetrating the blood-testis barrier (BTB), allowing transduction of haploid germ cells located within the seminiferous tubule lumen—such as round spermatids and spermatozoa—as well as spermatogonial stem cells (SSCs) residing adjacent to the basement membrane. Among these, AAV9 exhibited slightly higher transduction efficiency and maintained high cellular specificity without inducing significant inflammatory responses or tissue damage. AAV1 also showed a strong affinity for SSCs; although its depth of transduction was somewhat less than that of AAV9, it demonstrated a more uniform distribution across the seminiferous epithelium. In contrast, AAV8 was predominantly confined to the interstitial compartment of the testis. Its tropism was largely restricted to Leydig cells and, to a lesser extent, interstitial fibroblasts. Watanabe et al. confirmed that AAV8 is unable to cross the intact BTB, which limits its utility in targeting germline cells. As such, AAV8 is more suitable for gene delivery strategies aimed at correcting Leydig cell dysfunction or steroidogenic defects—such as those found in Lhcgr mutation-related DSD or male hypogonadism—rather than for interventions targeting the germline itself (Watanabe et al., 2018).

A notable preclinical application of AAV8-based therapy was reported by Xia. In their study, Lhcgr-deficient (Lhcgr−/−) mice, which mimic human Leydig cell failure (LCF), received interstitial injections of AAV8 encoding a functional Lhcgr gene. The therapy successfully restored Leydig cell steroidogenic function, leading to increased serum testosterone, recovery of sexual development, and partially restored spermatogenesis. Importantly, IVF using sperm from treated mice produced healthy offspring, confirming restoration of fertility (Xia et al., 2022). To overcome the limitations of AAV8, Zhang screened a panel of AAV serotypes and identified AAVDJ, a synthetic chimeric serotype, as a highly efficient vector for testicular gene delivery. Following intratesticular injection of AAVDJ-Lhcgr, they observed markedly improved transduction of Leydig cell progenitors in Lhcgr−/− mice. This led to significantly higher testosterone production compared to AAV8, complete restoration of testis morphology, and most critically, natural fertility restoration, with treated male mice siring fertile second-generation progeny through natural mating (Zhang et al., 2024). The therapy was well-tolerated, and no off-target expression was detected in liver, kidney, or spleen tissues, highlighting its specificity.

The choice of AAV serotype, delivery route (e.g., interstitial vs. intratubular), and target cell population (germ cells vs. somatic cells) greatly influences therapeutic outcomes. For instance, AAV1 and AAV9 may be preferable for gene delivery to germline cells due to BTB permeability, whereas AAV8 and AAVDJ are ideal for Leydig cell-specific expression (Watanabe et al., 2018; Zhang et al., 2024). These studies collectively suggest that AAV-mediated Lhcgr gene replacement may serve as a promising therapeutic modality for treating Leydig cell dysfunction-associated male infertility and DSD subtypes characterized by testosterone deficiency.

### 3.4 Lentiviral platforms for stable gene silencing and gonadal lineage induction in DSD

Lentiviruses belong to the retroviridae family and are enveloped viruses capable of stably integrating their genetic material into the host genome. Compared to other viral vectors such as AAV and adenovirus, lentiviral vectors offer several distinct advantages, including the ability to transduce non-dividing cells, high transduction efficiency, long-term gene expression, and a relatively large packaging capacity (up to 8–10 kb) (Naldini, 2015). These properties make lentiviruses among the most widely used vectors in gene therapy and cellular engineering, particularly suitable for sustained transgene expression or gene complementation in low-proliferation cell types such as neurons, hematopoietic stem cells, and induced pluripotent stem cells (iPSCs).

In another study focusing on fetal gonadal development, Macdonald et al. employed lentiviral-mediated gene silencing to investigate the role of Doublesex and Mab-3 Related Transcription Factor 1 (DMRT1) in maintaining testicular identity. The team constructed a third-generation SIN-LV vector carrying a shRNA expression cassette specifically targeting the mRNA sequence of human DMRT1. To monitor infection efficiency and tissue specificity, the virus also co-expressed GFP. The vector was microinjected into testis tissue slices from 12 to 15-week human fetuses and cultured *ex vivo*. DMRT1 knockdown resulted in disrupted Sertoli cell arrangement, downregulation of BTB-associated proteins such as CLDN11, and incomplete regression of Müllerian duct structures—hallmarks of disrupted AMH function. These phenotypes effectively recapitulate early features of PAIS and represent the first report of successful lentiviral gene silencing in human fetal testicular tissue. This study provided direct functional evidence for DMRT1’s critical role in maintaining testicular fate and demonstrated the tissue-specific and stable knockdown capabilities of lentiviral systems in primary fetal tissue (Macdonald et al., 2018).

Expanding on these approaches, Gonen et al. developed a multi-factor reprogramming system using lentiviral delivery to generate gonadal mesenchyme-like cells *in vitro*. This system was based on hiPSCs and involved the expression of five key sex-determining transcription factors: SOX9, GATA4, NR5A1, WT1, and DMRT1. Various combinations of these factors were tested, with the triplet of SOX9 + GATA4 + NR5A1 showing the most robust lineage-specific differentiation. Within 7–10 days post-transduction under defined induction conditions, the hiPSCs expressed high levels of gonadal markers including CYP17A1, AMH, and INSL3. Some cells exhibited classic Sertoli cell features (CLDN11-positive) and secreted steroid hormones. This lentivirus-based co-expression platform outperformed traditional methods such as plasmid electroporation in both efficiency and duration of expression, rendering it particularly suitable for long-term induction of gonadal lineages. The system holds promise for *in vitro* DSD modeling, high-throughput drug screening, and phenotype rescue experiments. In the future, it may also be integrated with CRISPR/Cas9-based gene repair strategies to support precise therapeutic interventions for DSD (Gonen et al., 2023).

It is important to note that despite their advantages in long-term expression, lentiviral vectors carry a risk of insertional mutagenesis. Riis emphasized that future clinical translation should employ third-generation SIN-LV and incorporate lineage-specific or inducible promoters to enhance safety (Lundgaard Riis and Jørgensen, 2022).

In summary, the application of gene therapy in DSD can be understood as a stepwise workflow rather than isolated technological attempts. In addition, various gene-editing tools and viral vectors—such as AAV, lentivirus, CRISPR/Cas9, TALENs, and ZFNs—have been applied in DSD-related models with distinct advantages and limitations (Table 2). The process typically begins with genetic diagnosis and variant identification, integrating phenotypic evaluation with sequencing to establish causative mutations or regulatory defects. This is followed by therapeutic strategy design, which may involve gene repair for point mutations, gene replacement for loss-of-function variants, or targeted regulation of endogenous gene expression. Once a therapeutic approach is defined, vector and delivery system selection becomes essential, with choices depending on target cell type, tissue specificity, and duration of expression. The designed strategies then require preclinical validation using pluripotent stem cell-derived gonadal cell models and relevant animal systems to assess efficacy, safety, and durability. Finally, all interventions must be evaluated within a framework of ethical and translational considerations, particularly in the pediatric and germline contexts, recognizing that current progress remains largely preclinical.

**TABLE 2:** Gene therapy tools/vectors in DSD–applications, advantages, and limitations.

Technology/Vector	DSD-related applications	Advantages	Limitations
AAV (AAV8/9)	Delivery of genes such as SRY, NR5A1, FOXL2, LHCGR	High safety profile, long-term expression, strong tissue specificity	Limited packaging capacity; risk of neutralizing antibodies and off-target expression
Lentivirus (LV)	Long-term expression and functional studies of genes like SOX9, WT1	Stable genomic integration, suitable for non-dividing cells, large gene capacity	High risk of insertional mutagenesis; requires stringent clinical safety evaluation
CRISPR/Cas9	Precise editing and correction of genes like SRY, SOX9, NR5A1, FOXL2	High precision, convenient operation, supports multiplex targeting	Off-target effects, potential toxicity due to DNA double-strand breaks
TALENs	Correction of DSD-related point mutations (e.g., SOX9, SRY)	High sequence specificity, ideal for precise single-nucleotide editing, no PAM requirement	Time-consuming construction, low transfection efficiency, limited to single-site edits
ZFNs	Targeted correction of DSD-associated regions such as WT1, SRY regulatory elements	Accurate DNA sequence recognition, suitable for functional element knockout or promoter reconstruction	Complex protein engineering, efficiency highly dependent on target design, off-target risk is less predictable

## 4 Challenges in gene therapy for DSD

### 4.1 Challenges associated with gene therapy

Despite the remarkable advancements in gene therapy technologies in recent years—particularly in the treatment of hereditary conditions such as DSD—their current clinical application remains hampered by multiple technical challenges. These include off-target effects, host immune responses, limited durability of transgene expression, and the risks associated with vector genome integration.

First and foremost, off-target activity remains a major safety concern, especially in CRISPR/Cas9-based editing systems. Degagné demonstrated that, even with optimized guide RNAs (gRNAs) and high-fidelity Cas9 variants such as HiFi-Cas9, unintended DNA cleavage events can still be detected *in vitro*. These off-target edits have the potential to activate apoptotic or oncogenic pathways, posing significant risks for therapeutic safety and efficacy (Degagné et al., 2024).

Secondly, immunogenicity constitutes another substantial barrier. Zittersteijn et al. highlighted that both AAV vectors and exogenous Cas proteins can elicit host immune responses shortly after administration. These include the generation of neutralizing antibodies that diminish gene transfer efficiency and may even induce systemic immune reactions (Zittersteijn et al., 2021). Kohn further noted that pre-existing anti-AAV antibodies in a subset of patients can render gene therapy ineffective at the initial dose (Kohn et al., 2023). Moreover, cytotoxic T lymphocyte-mediated clearance of successfully edited cells may further compromise long-term therapeutic benefit.

Third, the lack of sustained transgene expression poses a critical limitation to durable treatment outcomes. Jogalekar reported that gene expression from viral vectors such as AAV and lentivirus often diminishes over time due to factors including chromatin remodeling, host epigenetic regulation, and the proliferative dynamics of target cells (Jogalekar et al., 2022). Although the use of endogenous promoters and tissue-specific enhancers has shown promise in enhancing expression longevity, these strategies still fall short of the demands for chronic conditions like DSD that require prolonged therapeutic effect.

Lastly, the risk of vector integration into the host genome remains a major biosafety concern. Although AAV is classified as a non-integrating vector, Gonçalves reported that high-dose administration may still lead to rare random integrations, particularly in proliferative tissues, raising concerns about insertional mutagenesis and oncogenesis (Zittersteijn et al., 2021). In contrast, lentiviral vectors—despite their stable expression—pose a greater risk of insertional mutations due to their inherent integration properties, complicating their regulatory approval for clinical use (Fischer, 2023).

To address these limitations, multiple innovative strategies are currently under investigation. These include the development of high-fidelity Cas9 variants (e.g., SpCas9-HF1, eCas9), alternative viral vectors such as adenovirus, non-viral delivery platforms like lipid nanoparticles (LNPs), and epigenetic modulation techniques such as CRISPR interference (CRISPRi) or activation (CRISPRa). Additionally, the use of dead Cas9 (dCas9)-based epigenome editing, RNA-guided base editing, and single-stranded oligonucleotide donors (ssODNs) for homology-directed repair are gaining traction as safer and more precise therapeutic options (Liao et al., 2024; Hu et al., 2025). At present, no interventional clinical trials specifically evaluating gene therapy for DSD are registered. This reflects the field’s current stage as predominantly preclinical, underscoring the need for translational studies bridging animal models and future clinical applications.

### 4.2 Ethical considerations in gene therapy

The potential application of gene therapy in individuals with DSD raises a wide range of ethical concerns. These complexities are not only rooted in the inherent risks of biomedical technologies but also stem from intersecting issues involving gender identity, patient autonomy, intergenerational genetic safety, and sociocultural values.

First, the introduction of exogenous genes—particularly in germline cells—poses a permanent impact on future generations and remains one of the most contentious ethical debates. Although most current gene therapy strategies focus on somatic interventions, many phenotypes associated with DSD emerge during early embryogenesis, making germline editing theoretically more effective. However, the international scientific and ethical communities have adopted a cautious stance. Organizations such as UNESCO and the World Health Organization have called for a moratorium on clinical germline editing in humans until sufficient consensus is reached regarding its technical reliability, safety, and societal implications (World Health Organization, 2021).

Second, the implementation of informed consent presents significant challenges. Most DSD patients are children, adolescents, or even neonates, whose gender identity is not yet fully established at the time of diagnosis or treatment. Making irreversible medical decisions—such as gene-based interventions—without the patient’s direct authorization raises serious ethical concerns. Researchers emphasized that irreversible treatments in infants with DSD should be deferred until the individual reaches an age capable of informed decision-making (Diamond et al., 2018). Similarly, Barseghyan stressed that gene therapy should not reinforce gender-normative biases but instead prioritize functional restoration, such as gonadal function, hormone production, and fertility potential (Barseghyan et al., 2015). Embryonic and reproductive ethics further complicate the discussion. Some experimental approaches propose gene correction at the embryonic stage to prevent the manifestation of DSD phenotypes (Kohn et al., 2023). However, such interventions involve direct manipulation of unborn life and have provoked public concern about “designer babies” and eugenic practices.

Finally, issues of patient privacy and genetic data usage must not be overlooked. DSD involves highly sensitive information related to reproductive health and gender identity. If genomic data were to be used for purposes beyond research or clinical care without proper safeguards, it could lead to stigmatization or social discrimination. Arboleda advocated for stringent encryption and anonymization of genetic and identity-related data for DSD patients, especially in public registries such as the I-DSD Registry. They also emphasized the necessity of explicit, individualized consent before any data sharing (Arboleda et al., 2014).

## 5 Future directions

While significant advances have been made in gene therapy applications for DSD, several areas remain under active investigation and development. These include the refinement of delivery systems, enhancement of editing specificity, and development of suitable animal models.

One key future direction is the development of tissue-specific delivery systems that enhance gene transfer efficiency while minimizing off-target expression. Recent studies have explored engineered AAV capsids with enhanced tropism toward testicular somatic cells or gonadal precursor lineages (Liao et al., 2024). Lipid nanoparticles (LNPs) with modified surface ligands have also shown promise in mediating organ-selective delivery, particularly for transient mRNA expression without genomic integration (Hu et al., 2025). Further efforts should aim to combine capsid engineering, microRNA-regulated cassettes, and route optimization to improve spatial specificity of gene therapy in DSD.

Minimizing off-target effects in CRISPR-based systems also remains a critical objective. Although high-fidelity Cas9 variants such as SpCas9-HF1 and eSpCas9 have shown substantial improvement, complete elimination of unintended DNA cleavage is still not achievable (Degagné et al., 2024). Novel approaches including RNA-guided base editors and prime editing systems offer potential for safer interventions by avoiding double-strand breaks altogether. Combining these systems with real-time single-cell off-target screening technologies may yield robust strategies for clinical-grade applications in DSD.

Notably, the development and refinement of animal models represent a cornerstone for future preclinical validation. Current models include Sry-knockout mice for 46, XY gonadal dysgenesis, Sox9 enhancer deletion models for sex reversal, and Lhcgr-null mice for Leydig cell failure (Croft et al., 2018; Xia et al., 2022). However, many DSD phenotypes, especially those involving compound heterozygous mutations or regulatory element dysfunctions, are not fully recapitulated in existing rodent models. Generation of patient-derived xenografts (PDXs) using human iPSCs differentiated into gonadal cell types, or CRISPR-based multiplexed mutation mice, will likely provide more translational platforms. Moreover, larger animal models such as rabbits or pigs could offer advantages in reproductive anatomy and endocrine dynamics more analogous to humans.

Collectively, future research should emphasize the integration of vector design, editing precision, and disease modeling to accelerate the clinical translation of gene therapy for DSD. Interdisciplinary collaboration among molecular biologists, endocrinologists, and bioengineers will be essential to overcome current technical bottlenecks.

## 6 Conclusion

Gene therapy has shown emerging promise in the treatment of DSD, particularly through AAV-mediated gene delivery and CRISPR/Cas9-based genome editing. Preclinical models have demonstrated effective manipulation of key DSD-associated genes, including SRY, SOX9, NR5A1, WT1, FOXL2, and AR, using diverse vectors such as AAV8, AAVDJ, and lentivirus. These approaches have enabled restoration of gene function, correction of pathogenic variants, and modulation of gene expression in both *in vivo* and hiPSC-based systems. However, clinical translation remains limited by concerns regarding off-target effects, vector immunogenicity, transient expression, and potential insertional mutagenesis.

Moreover, ethical considerations—such as those related to germline modification, informed consent in pediatric patients, and long-term impact on sex identity—must be addressed alongside scientific progress. Future therapeutic development should prioritize precision editing, tissue specificity, and ethically guided clinical frameworks to ensure both efficacy and safety.
